# TRIM5α self-assembly and compartmentalization of the HIV-1 viral capsid

**DOI:** 10.1038/s41467-020-15106-1

**Published:** 2020-03-11

**Authors:** Alvin Yu, Katarzyna A. Skorupka, Alexander J. Pak, Barbie K. Ganser-Pornillos, Owen Pornillos, Gregory A. Voth

**Affiliations:** 1 Department of Chemistry, Chicago Center for Theoretical Chemistry, Institute for Biophysical Dynamics, and James Franck Institute, The University of Chicago, Chicago, IL 60637 USA; 2 Department of Molecular Physiology and Biological Physics, University of Virginia, Charlottesville, VA 22903 USA

**Keywords:** Computational biophysics, Computational chemistry, Molecular dynamics, Statistical mechanics

## Abstract

The tripartite-motif protein, TRIM5α, is an innate immune sensor that potently restricts retrovirus infection by binding to human immunodeficiency virus capsids. Higher-ordered oligomerization of this protein forms hexagonally patterned structures that wrap around the viral capsid, despite an anomalously low affinity for the capsid protein (CA). Several studies suggest TRIM5α oligomerizes into a lattice with a symmetry and spacing that matches the underlying capsid, to compensate for the weak affinity, yet little is known about how these lattices form. Using a combination of computational simulations and electron cryo-tomography imaging, we reveal the dynamical mechanisms by which these lattices self-assemble. Constrained diffusion allows the lattice to reorganize, whereas defects form on highly curved capsid surfaces to alleviate strain and lattice symmetry mismatches. Statistical analysis localizes the TRIM5α binding interface at or near the CypA binding loop of CA. These simulations elucidate the molecular-scale mechanisms of viral capsid cellular compartmentalization by TRIM5α.

## Abstract (web summary)

Tripartite-motif containing (TRIM) proteins modulate cellular responses to viral infection. Here the authors use molecular dynamics simulations to demonstrate that TRIM5α uses a two-dimensional lattice hopping mechanism to aggregate on the HIV capsid surface and initiate lattice growth.

## Introduction

Tripartite motif containing proteins (TRIMs) are a large family of proteins that play central roles in host cellular responses to viral infection^1–4^. TRIMs have been implicated in diverse biological processes, including autophagy, cell differentiation, apoptosis, and intracellular signaling^5–8^. Several TRIM proteins directly restrict viral activity by interfering with distinct steps in the viral life cycle, while others regulate signal transduction pathways induced by innate immune sensors. TRIM5α and TRIMCyp, in particular, have been found to be responsible for the species-specific resistance to infection by the human immunodeficiency virus type 1 (HIV-1) in rhesus macaques^9^ and owl monkeys^10^, respectively.

TRIM5α proteins are cytoplasmic proteins that bind to the retroviral capsid of HIV-1 and promote signaling pathways that alert the innate immune system to invasion^11,12^, causing the premature dissociation of the capsid and inhibiting reverse transcription^13–15^. Higher-ordered oligomers of TRIM5α spontaneously assemble into hexagonally patterned structures on the surface of the capsid, which are detectable under negative-stained electron microscopy^16,17^. Like all tripartite motif containing proteins, TRIM5α consists of three distinct domains – a RING domain, B-box domain, and coiled-coil domain (i.e., RBCC or tripartite motif). At low concentrations in solution, TRIM5α proteins primarily form dimeric complexes, mediated by interactions between two antiparallel α-helices that comprise the coiled-coil domain. The B-box domain caps the N-terminal end of each α-helix and mediates interactions between TRIM5α dimers. B-box domains interact with the B-box domains of other TRIM5α dimers in a layered fashion with electrostatic interactions sandwiching a hydrophobic core to produce a three-fold symmetric trimer-of-dimers structure^18^. In addition, TRIM5α contains a C-terminal SPRY domain, connected to the center of the coiled-coil domain by the L1 helix and a flexible linker region, that has a very weak affinity for the capsid protein (CA) (estimated *K*_D_ > 1 mM)^13,19–21^.

Given the low affinity of individual TRIM5α monomers for the capsid, how does TRIM5α effectively encage the viral capsid? Dimerization enhances TRIM5α binding in vitro, and several studies, including ours, have suggested that the weak interactions are amplified by avidity effects resulting from higher-ordered oligomerization, which positions the SPRY domains to interact with repeating structural elements on the capsid surface^22,23^; although none have explored the dynamics of how this might occur. In this work, we investigate the self-assembly mechanisms of TRIM5α on the HIV-1 retroviral capsid, using a combined coarse-grained (CG) molecular simulation, cryo-electron tomography (cryo-ET) imaging, and computational analysis approach.

## Results

### CG model development for TRIM5α and CA

Mature HIV-1 capsid structures are large protein assemblies consisting of ~1500 individual CA domains arranged in a fullerene cone around the viral RNA, reverse transcriptase, and other enzymes that facilitate the replication of the virus. At least two hundred TRIM5α monomers are required to form an encaging hexagonal lattice around the core of the virus that blocks HIV infection in a slow dynamical process that takes place on the order of minutes^24^ and is currently not amenable to analysis by all-atom molecular dynamics simulations.

**Fig. 1:**
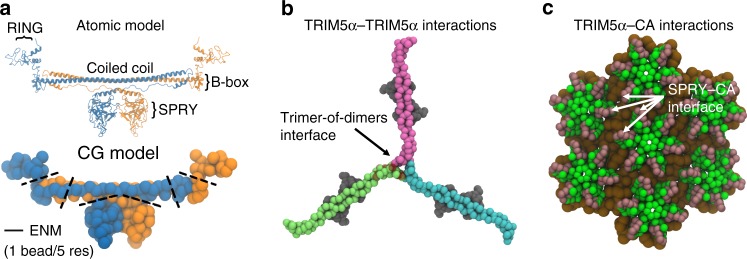
Minimal interactions for capsid-induced TRIM5α self-assembly. **a** Atomic and CG representations of the TRIM5α dimer. Each monomer subunit is colored in orange and blue, respectively. Labels indicate distinct structural domains (Top). Dashed lines highlight locations of elastic network models used to confer flexibility to the dimer in the CG model (Bottom). **b** A snapshot of a higher-ordered TRIM5α trimer-of-dimers structure assembled during our simulations. Dark red spheres depict the locations of virtual sites for the central TRIM5α dimer (pink), which act as tethering sites for the adjacent TRIM5α dimer (lime). **c** CG representation of the capsid surface. The N-terminal domain is colored in green, whereas the C-terminal region is colored in brown. Dark pink spheres reflect sites of attractive interactions with the SPRY domain.

To surmount the computational cost associated with simulations at an atomic level of detail, we developed a CG model for TRIM5α self-assembly on viral capsids, using methods established in prior studies^25–28^. An atomic model for TRIM5α was first constructed from crystallographic fragments available in the Protein Data Bank, and subsequently coarse-grained at a resolution of 1 CG particle per 5 amino acid residues (Fig. 1a). Elastic network models (ENM) were used at the interfaces of specific domains – SPRY–coiled coil, B-box–coiled-coil, and RING–B-box domains – to confer protein flexibility (Supplementary Fig. 1). At a minimum, two intermolecular driving forces are necessary for TRIM5α assembly on capsids: interactions between the dimers that mediate the higher-ordered assembly of the lattice (Fig. 1b), and interactions between dimers and CA that regulate the binding to the capsid surface (Fig. 1c). Attractive interactions between B-box domain residues were added to promote the formation of the crystallographically resolved trimer-of-dimer structures. Attractive interactions were added between SPRY and CA domain residues that demonstrated decreased or abolished TRIM5α binding in experimental mutagenesis studies^21,29^. (For a complete description on the models, see also Materials & Methods).

### Molecular mechanisms of TRIM5α self-assembly

**Fig. 2:**
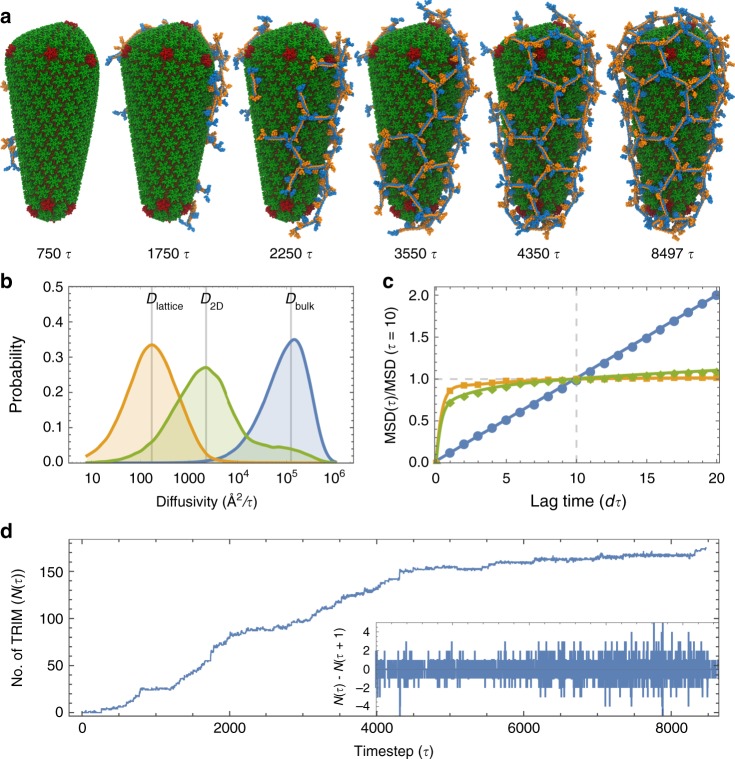
Coupled TRIM5α self-assembly and capsid binding. **a** The assembly process of TRIM5α shows how TRIM5α encages the viral capsid. Time points (*τ*) are given relative to the start of the simulation. **b** Normalized distribution of diffusivity values for TRIM5α dimers in bulk solvent (blue; *D*_bulk_ = 123,315 Å^2^/*τ*), TRIM5α dimers bound to the capsid surface (green; *D*_2D_ = 2224 Å^2^/*τ*), and TRIM5α lattices bound to the capsid (orange; *D*_lattice_ = 174 Å^2^/*τ*). **c** Mean-squared displacements (MSD) of the TRIM5α subunits as a function of the lag time in units of *τ*. The MSD(*t*) values were normalized by the MSD (*t* = 10*τ*). Line colors correspond to the populations denoted in **b**. **d** Growth in the number of TRIM5α subunits that form lattice contacts with other TRIM5α, on the capsid surface, *N*(*τ*), as a function of time, *τ*. Inset shows the finite difference of *N*(*τ*) for *t* = 4000–8000*τ*. Positive values indicate that lattice contacts form, whereas negative values indicate that lattice contacts break. Note that all units of time are given in *τ* = 250,000 CG simulation time steps.

TRIM5α has an intrinsic propensity to form hexagonal protein assemblies at high concentrations. Negative-stain electron microscopy experiments have demonstrated that purified TRIM5α spontaneously assembles into two-dimensional arrays composed primarily of hexagonal rings^16^. To gain insight into the self-assembly mechanisms of TRIM5α, we performed CG molecular dynamics simulations of TRIM5α in the presence of conical capsids derived from cryo-ET data on intact virions^30^. Snapshots of the assembly process taken at various stages of lattice formation are shown in Fig. 2a and Supplementary Movie 1.

In the simulations, TRIM5α dimers rapidly bind to and dissociate from the capsid. While the dimers are bound, they diffuse two-dimensionally across the capsid surface and contact other bound dimers at the B-box interface to form a central trimer-of-dimers structure. Formation of the trimer-of-dimer structure nucleates the growth of an initially hexameric lattice (*t* = 0–1483*τ*). Surprisingly, non-hexagonal defects also form in the TRIM5α lattice at regions of high CA curvature. Pentameric defects form primarily along the broad end of the conical capsid (*t* = 1699*τ*); a tetrameric defect forms at the narrow end of the capsid (*t* = 4297*τ*); and a heptameric defect forms at the base of the broad end, adjacent to three pentameric defects (*t* = 4501*τ*). Alternating pentamer–heptamer defect motifs lower the energetic strain of defect incorporation in the lattice.

Large-scale reorganization of the lattice occurs as the entire lattice shifts to accommodate the addition of TRIM5α onto the growing lattice (*t* = 1219*τ*, 4171*τ*, 5125*τ*). Intermediate defect structures that are less stable at particular positions on the lattice, break and reform into more stable oligomers. A metastable pentamer later converts into a hexamer (*t* = 1219*τ*), and an initially tetrameric oligomer in the lattice converts into a pentamer (*t* = 5125*τ*). The resulting lattice consists of 16 hexamers, 12 pentamers, 2 heptamers, and 1 tetramer, and has an Euler’s characteristic that is the same as that of enclosed fullerene structures (*χ*_TRIM_ = 2).

Closer inspection of the association and dissociation of TRIM5α with the capsid surface (Supplementary Movie 2) revealed that the dimers diffuse across the surface by “hopping” across metastable SPRY–CA domain interaction hotspots. There are two SPRY domains per dimer, and as contact between one of the SPRY domains and the capsid breaks, the dimer can rotate about the anchoring SPRY domain before binding at another SPRY–CA interaction hotspot, allowing for diffusive movement that resembles non-directional step-by-step “walking”. To quantify this 2D diffusive behavior, we measured the diffusivities for three distinct subpopulations of TRIM5α throughout the assembly process: (1) dimers in bulk solvent (*D*_bulk_), (2) dimers that bound and did not contact any other dimers (*D*_2D_) and (3) dimers that bound and made lattice contacts with other TRIM5α associated to the capsid (*D*_lattice_). The peaks of the diffusivity distributions yield the diffusion coefficients, which follow the expected trend: *D*_lattice_ < *D*_2D_ < *D*_bulk_ (Fig. 2b).

The mean-squared displacements (MSDs) calculated for dimers in bulk solvent correlated linearly with the lag time, consistent with the mobility of freely diffusing particles (Fig. 2c). Dimers bound to the capsid however, had MSDs that plateaued to a constant value, characteristic of constrained diffusion, with dimers that formed TRIM5α lattice network contacts plateauing faster than dimers that did not (decay rate: *λ*_lattice_ = 2.23; *λ*_2D_ = 1.16). The converged MSDs, indicative of the size of the constraining region (*s*), also suggested that lattice oligomers diffusing on the capsid surface were more highly constrained than individual dimers (*s*_lattice_ = 203 Å^2^; *s*_2D_ = 3,297 Å^2^).

Growth of the encaging TRIM5α lattice can be characterized as a stochastic process in which the continual formation and breakage of contacts between dimers in the lattice is necessary to drive the process towards hexagonal assembly. We tracked how the number of lattice-forming monomers, *N*(*τ*), varied with respect to the simulation time (Fig. 2d). The concentration of free TRIM5α in solution provides the chemical driving force for the assembly process, and was at the start of the simulation initially set to 3.2 μM, consistent with the TRIM5α concentration used in in vitro cryo-EM experiments^17^. As assembly proceeds, the rate of lattice growth slows, most apparently from *t* = 5000–8471*τ*, in part as a result of the drop in the concentration of free TRIM5α in the simulation system, and the decrease in available capsid surface area that is unoccupied by TRIM5α. Intriguingly, the growth of the lattice is interspersed with periods of rapid growth and stalled intermediates, most notably at *t* = 913–1219*τ*, 1699*τ*, and 4171*τ*. As discussed earlier in this section, the time points *t* = 1219*τ* and 4171*τ* involve lattice reorganization events that precede continued growth, and *t* = 1699*τ* involves the formation of pentameric defects at the broad end of the capsid that stabilizes the curved end of the TRIM5α lattice. The finite difference of *N*(*τ*), in which negative values correspond to the number of TRIM5α contacts formed and positive values correspond to the number of TRIM5α contacts broken at each timestep, *τ*, indicates that on average two TRIM5α contacts in the lattice are formed or broken at each timestep. These results suggest that the coexistence of assembly and disassembly of the lattice as it grows and wraps around the capsid, or dynamic instability, is essential to the encaging process.

### Competing protein interactions determine assembly behavior

**Fig. 3:**
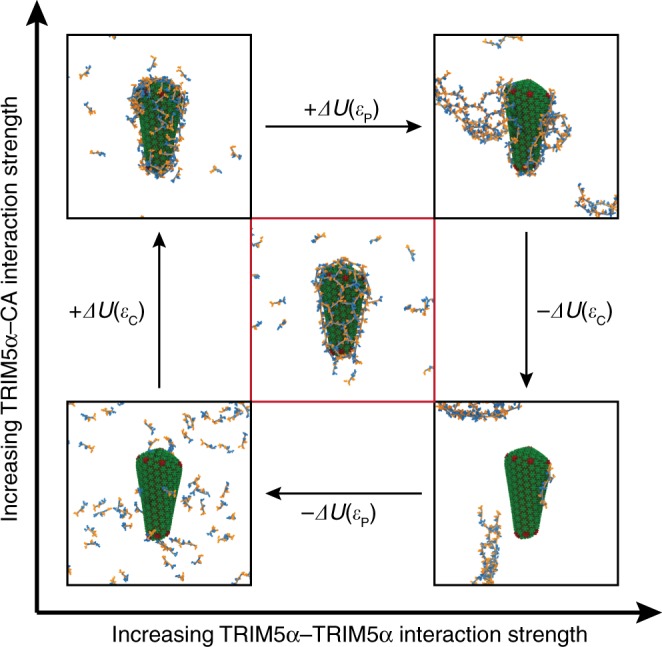
A thermodynamic cycle depicts the phase behavior of TRIM5α lattice assembly. *ε*_P_ and *ε*_C_ are parameters that tune the strengths of interactions between the protein–protein (TRIM5α–TRIM5α) contacts of TRIM5α and the protein–capsid (TRIM5α–CA) contacts, respectively, in the potential energy. Panels are CG assembly simulations that proceeded to completion (~10,000 *τ*) for: *ε*_P_ = 0.5, *ε*_C_ = 0.3 kcal/mol (Bottom Left); *ε*_P_ = 1.2, *ε*_C_ = 0.3 kcal/mol (Top Left); *ε*_P_ = 1.2, *ε*_C_ = 1.0 kcal/mol (Top Right); *ε*_P_ = 1.2, *ε*_C_ = 0.3 kcal/mol (Bottom Right) and *ε*_P_ = 0.7, *ε*_C_ = 0.35 kcal/mol (Center). Hexagonal assembly of TRIM5α is critically balanced between *ε*_P_ and *ε*_C_.

The strength of the molecular interactions between TRIM5α dimers (TRIM5α–TRIM5α; *ε*_P_) and between TRIM5α dimers and the capsid (TRIM5α–CA; *ε*_C_) compete to determine the overall assembly behavior. We performed multiple CG simulations, in which the attractive interactions at the B–box domain interface were varied from *ε*_P_ = 0.5–1.2 kcal/mol and attractive interactions between the SPRY and CA domains were varied from *ε*_C_ = 0.3–1.0 kcal/mol. Endpoints of the simulations (Fig. 3) show markedly different assembly behavior as a consequence of the relative strengths of the TRIM5α–TRIM5α and TRIM5α–CA interactions.

When both interaction strengths are below a critical value (*ε*_P_ = 0.5 kcal/mol; *ε*_C_ = 0.3 kcal/mol), TRIM5α dimers are diffusive in solution and neither self-assemble nor bind to the capsid. At low TRIM5α–TRIM5α and high TRIM5α–CA interaction strengths, the dimers bind to the capsid, but do not interact with one another on the capsid surface, and global hexagonal lattice formation is not present. When both interactions are too strong (*ε*_P_ = 1.2 kcal/mol; *ε*_C_ = 1.0 kcal/mol), the dimers bind and form assemblies that grow and protrude from the capsid. At high TRIM5α–TRIM5α and low TRIM5α–CA interaction strengths, the dimers aggregate into large assemblies in solution that grow until the free dimer concentration in solution is exhausted, and do not encase the capsid.

Hexagonal assembly of the TRIM5α lattice on the capsid is only present over a narrow range of interaction strengths (*ε*_P_ = 0.6–0.7; *ε*_C_ = 0.35–0.4 kcal/mol) and sensitive to small perturbations in the interaction energetics. Relatively weak interactions between the SPRY domain and capsid are required for the growing lattice to reorganize and for TRIM5α dimers to diffuse on the capsid; otherwise lattice configurations become kinetically trapped in states where bound dimers do not interact with other neighboring dimers (Fig. 3; Top Left panel). Weak interactions between dimers are also required to prevent the rapid growth of TRIM5α aggregates in solution and allow defects to adopt more stable hexagonal configurations (Fig. 3; Bottom Right panel). Interaction ranges that facilitated hexagonal assembly on the lattice had stronger interactions between the dimers than between the SPRY and CA domains. Notably, TRIM5α dimers do not self-assemble in bulk solution for these interaction strengths (e.g. see Supplementary Fig. 2, *ε*_P_ = 0.7; *ε*_C_ = 0 kcal/mol), indicating that nucleation on the capsid surface, mediated by SPRY–CA domain interactions, is required to trigger lattice assembly at these concentrations. Hexagonal assembly of the lattice thus appears to be tuned between these two competing factors.

### Non-hexagonal defects form in TRIM5α lattices

The simulations show that non-hexagonal defects can form in TRIM5α lattices to alleviate strain induced by curvature of the capsid. For viral HIV-1 CA protein, exactly 12 pentameric defects are required to form enclosed capsid cores, consistent with those seen in ideal fullerene structures^31,32^. In vitro experiments have also demonstrated that CA can form helical tubes without defects^33–35^, since hexagonal lattices have an Euler’s characteristic (*χ*_Hex_ = 0), which matches the topological structure of cylindrical tubes.

**Fig. 4:**
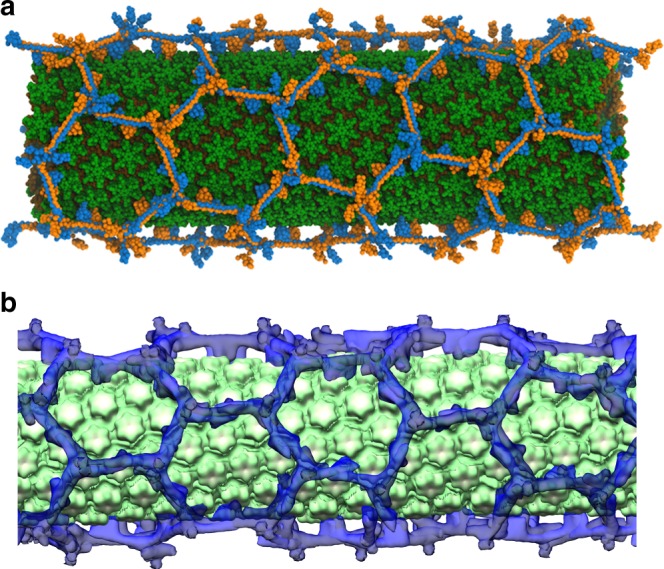
TRIM5α assembles into hexagonal lattices on capsid tubes. **a** Simulation endpoint for the TRIM5α assembly process on a cylindrical surface. **b** Electron cryotomography (cryo-ET) reveals that TRIM5α forms hexagonal lattices on the surface of CA tubes.

We initially hypothesized that TRIM5α lattices would also form purely hexagonal lattices on CA tubes. We expressed and purified TRIM5α and HIV-1 CA proteins and then co-incubated them at low ionic strength conditions to produce CA tubes that were extensively bound with TRIM5α^17,18^, which were subsequently imaged by electron cryotomography. We then performed sub-tomogram analysis on a single tube, focusing on each of the repeating units (HIV-1 CA hexamer, TRIM5a dimer, and TRIM5a trimer), which yielded 20–30 Å reconstructions. Mapping back these averages to the original tomogram, using Euler angles and positions derived from sub-tomogram averaging^36^, allowed us to visualize the relative arrangements of the repeating units^37^. This analysis showed that the tube was composed of an inner array of CA hexamers surrounded by ring-like decorations formed by TRIM5α (Fig. 4b).

To our surprise, the experimental TRIM5α lattice map contained multiple defects including pentameric and heptameric TRIM5α rings. The defects formed seam-like regions, or grain boundaries, along the tube, and clustered to form alternating pentamer and heptameric defect motifs (see Supplementary Fig. 3). A segment of the helical CA tube was extracted from the lattice map, which was then simulated with TRIM5α added in bulk solvent. The CG simulations resulted in both defect-free TRIM5α lattices (Fig. 4a), and lattices that contained pentameric and heptameric defects. Defect formation depended highly on the growth of the TRIM5α lattice. Symmetry mismatches between the TRIM5α lattice and the underlying CA lattice also cause defect incorporation. Defects are inherently less stable compared to regular hexagonal states, and the presence of these defects indicate that they were kinetically trapped during lattice growth. Qualitatively, the hexagonal regions of the TRIM5α lattices directly imaged by cryo-ET and predicted from our CG simulations were in good agreement (Fig. 4).

**Fig. 5:**
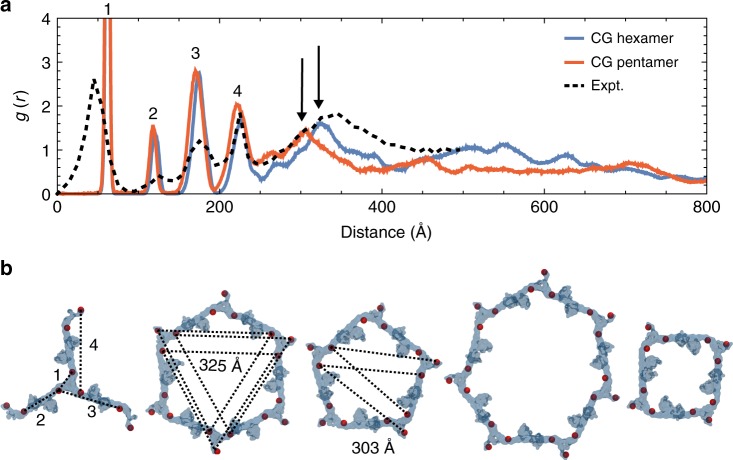
Non-hexagonal defect structures of TRIM5α lattices. **a** Pair correlation functions, *g*(*r*), between the virtual sites’ center-of-geometry as a function of the radial distance (Å), calculated for TRIM5α subunits that form hexagonal ordered lattices (blue) and pentagonal ordered lattices (red) on the capsid. The black dashed lines indicate the *g*(*r*) calculated from models derived from experimental cryo-ET densities (see Fig. 4). Peaks 1–4 are labeled. Arrows indicate close-range differences in the lattice spacing peaks between hexagonal and pentagonal lattices. **b** Higher-ordered TRIM5α structures that spontaneously assemble on the capsid during simulation. TRIM5α subunits are colored in transparent blue, and the center-of-geometry for the virtual sites is depicted as red spheres. Dotted lines indicate intermolecular distances that form peaks in the pair correlation function, *g*(*r*). Source data are provided as a Source Data file.

To quantitatively compare the hexagonal lattice structure and defect structures predicted from our CG simulations (Fig. 5b) to that derived from cryo-ET, we computed pair correlation functions, *g*(*r*), (Fig. 5a) with respect to the center-of-geometry of B-box domain residues. The *g*(*r*) measures how the density of particles varies as a function of distance, and peaks at regular spacings in structures with symmetry. Peak 1 corresponds to the trimer-of-dimers interface, which is smaller in the cryo-ET lattice (~43 Å) than that of the simulated lattices (~63 Å), indicating that the interface may be more compact than predicted by our simulations. The positions of peaks 2–4 agree between the experimental and simulated lattices; there are slight differences between the hexameric and pentameric lattices, which may reflect distortions in the trimer-of-dimers, as they adopt angular distributions of 120° and 108°, respectively. Hexameric lattices contain a peak at 325 Å that is downshifted to 303 Å in pentameric lattices, which corresponds to the smaller size of the pentamers. The experimental cryo-ET lattice is peaked at both 303 Å and 325 Å, since it contains both hexamers and pentamers. The pair correlation functions were not calculated for heptameric nor tetrameric defects, since only one or two of each formed in each CG trajectory, which did not allow for enough statistics to converge the *g*(*r*). Tetrameric structures occupied regions of high CA curvature like that of the narrow end of the capsid, and were the most energetically unstable during simulation. Tetrameric defects were not detected in cryo-ET images of TRIM5α associated CA tubules, which are less curved than capsids. In a prior study, cryo-ET imaging of TRIM5α associated with capsids stabilized by disulfide-crosslinks reveals the presence of defects that resemble tetramers; although the resolution is too poor for conclusive validation^17^. We speculate that the tetrameric defects observed in the simulations are transient metastable states. The computationally predicted pentameric and heptameric structures, are in good agreement with the cryo-ET experiments. Importantly, our CG simulations independently predicted the existence of pentameric and heptameric defects prior to experimental validation.

### SPRY–CA binding interface

The SPRY domain affinity to CA is so weak that it is difficult to measure experimentally (estimated *K*_D_ > 1 mM)^13,19–21^, and thus far has proven refractory to crystallization. TRIMCyp is a related RBCC protein found in owl monkeys that contains Cyclophilin A (CypA) in place of the SPRY domain and also blocks HIV-1 infection. CypA has a much higher affinity for the CA protein (*K*_D_ ~ 10 μM)^38–40^, and the high-resolution crystallographic structure of CypA in complex with CA reveals that it binds to a flexible loop region that connects helices 4 and 5 (resid 80–100)^41^. Given the weak affinity between the SPRY and CA domains and the lack of available structural data, several studies have attempted to model the SPRY binding interface either using computational methods^42,43^ or experimental mutagenesis^29,44^. Our CG simulations provide an alternative approach.

**Fig. 6:**
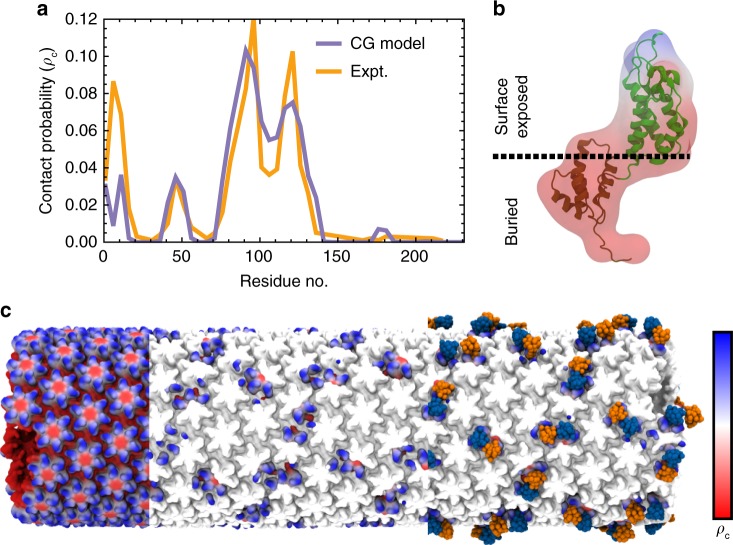
The SPRY domain binding interface. **a** Probability for residues on the CA domain to form contacts with TRIM5α calculated from both simulation (CG) and experimental cryo-ET density maps (Expt). **b** The simulated contact probability is shown in a 3D map overlaid with the atomic structure of the CA domain and colored by regions from highest contact probability (blue) to lowest contact probability (red). **c** The CG structure of the CA tubule with highest contact probability (*p*_c_ = 0.10) in blue and regions of lowest probability colored in red (*p*_c_ = 0.0) sub-divided in three sections. The leftmost section shows the contact probability across the tubule; the middle section shows the probability at positions for which the SPRY domain bound during a CG assembly simulation; the rightmost section shows the binding positions of the SPRY domains. Source data are provided as a Source Data file.

To locate the SPRY–CA binding interface, we calculated a residue-specific contact probability map for the CA domain. During the assembly simulations, CA residues within 20 Å of the SPRY domains were tabulated and used to compute a 1D probability distribution for contact. The same calculation was also performed using a TRIM5α and CA model fit to the experimental cryo-ET densities (Fig. 6a). The simulated and experimental contact probabilities agree well; although the experimental probability distribution has sharper peaks, in part because it was calculated from a single cryo-ET structure. Both probability distributions indicate the highest occupancy for SPRY in the CypA binding region and the adjacent loop region between helices 6 and 7 (resid 115–130). Lower contact probabilities were found for the β-hairpin region at the entrance to the central pore of the helix (resid 1–15), recently hypothesized to change conformation in response to protonation^45^, and a more buried loop region between helices 2 and 3 (resid 40–50). The elevated contact probabilities in these regions may be attributed to steric contact, once the variable loops of the SPRY domain contact the CypA binding region; note that the cryo–ET structure has a higher occupancy for the β-hairpin region than that of the computational results, indicating the SPRY domain may have a slight attractive interaction in that region, which was not captured in our CG simulations. Interestingly, the contact probabilities for CA residues matches well with those derived from the experimental densities for both residues that had attractive interactions with the SPRY domain (resid 83–132) and residues that did not, likely as a combination of sterics, excluded volume, sites that facilitated TRIM5α lattice contacts, and the attractive TRIM5α–CA potential. Overall, however, the agreement between the simulated and experimental contact probabilities further validates our CG simulations.

The contact probabilities were mapped back onto volumetric surfaces for CA (Fig. 6b, c). Highlighting the surfaces on the CA tube in which the SPRY domain appeared bound during assembly (Fig. 6c) reveals that TRIM5α binds to substantially varied interfaces on the capsid surface, including along two-fold, three-fold symmetry axes between hexamers^29^, and above the NTD of the CA domain. Our results suggest TRIM5α binds primarily at or near the CypA binding loop of HIV-1 CA, which is consistent with recent nuclear magnetic resonance spectroscopy results that demonstrate differences in the chemical shifts of residues in this region upon SPRY binding^46^.

### TRIM5α binds to multiple capsid morphologies

**Fig. 7:**
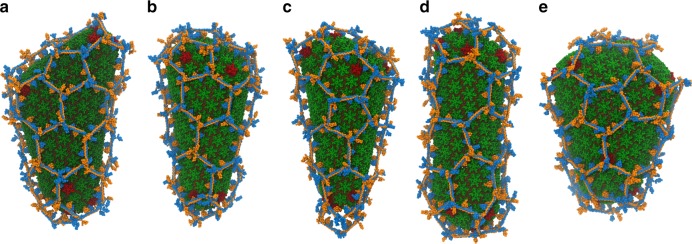
TRIM5α binds to multiple capsid morphologies. **a**–**e** Note the presence of pentameric and heptameric defects. CG capsid morphologies were derived from directly fitting cryo-ET data of the core structure collected from intact virions^30^.

HIV capsids are pleomorphic structures that adopt several distinct morphologies to package viral RNA^30,47^. It is possible that TRIM5α forms either incomplete lattices or lattices that contain differing structures and defects in response to the variable curvatures found in these capsids. To address this, we constructed CG models for five other morphologies of viral capsids, based off the prior experimental models developed by imaging the capsid structure within intact virions using cryo-ET^30^. Assembly simulations were performed until the capsids were completely encased by the TRIM5α lattice (Fig. 7). The overall lattice structures and defects formed were not significantly different among the capsid morphologies. Hexameric, pentameric, and heptameric structures were present in all cases. Two of the capsid structures had very narrow ends, which caused a tetrameric defect and a puckered hexamer to form (Fig. 7b, c). Assembly on two other capsid morphologies resulted in slightly incomplete lattices on the broad ends of the capsid (Fig. 7d, e).

## Discussion

Taken together, our simulation results suggest a surprisingly rich and complex dynamical picture of how TRIM5α encages the viral capsid core to block HIV-1 infectivity. Presently, simulation is indispensible for studying such dynamical effects since experimental techniques cannot yet provide this information. Diffusive motion of individual dimers on the capsid surface leads to a dynamical growth of the lattice, in which continual assembly and disassembly of the lattice is necessary to drive the encaging process. We predicted and experimentally validated the existence of pentameric and heptameric defects, and suggest that metastable tetrameric defects may also form in regions of high CA curvature. These defects are required to enclose the capsid and may allow the TRIM5α lattice to adapt to the varied capsid structures found in pleomorphic viruses like HIV. Since retroviruses apply strong selective pressures on their host elements, we speculate that the high sensitivity in the competing interactions that determine assembly (between dimers and between individual dimers and CA) indicates they are finely tuned by evolution for encaging the viral capsid.

Do other TRIM proteins self-assemble in this fashion? The answer so far, is unclear. Given the high conservation of the B-box motif compared to the SPRY domain, host cells seem to have adapted a modular response in the TRIM system, whereby distinct SPRY domains can be swapped to provide specific affinity for targets. These targets bind to the variable loops of the SPRY domain. TRIM21 is an autoantigen, for example, whose SPRY domain binds the IgG antibody with high affinity^48^, and can reportedly neutralize IgG-coated viral capsids^49,50^. Mutations in the variable loops of the SPRY domain of TRIM20 cause genetic disorders such as Familial Mediterranean Fever^51^. TRIM28 mediates transcriptional silencing of murine leukemia virus^52,53^. Furthermore, TRIM43 is an antiviral protein that has been recently reported to specifically block herpesvirus infection^54^.

The diversity of biological roles these TRIM proteins play, from autophagy to cell differentiation, implicates a general function for these proteins in cellular compartmentalization and scaffolding. Investigations into the mechanisms of these proteins may inform general principles towards the design of self-assembling, encaging nanostructures.

## Methods

### CG protein models

The initial atomic model for the TRIM5α monomer was constructed by assembling crystallographic fragments of the RING (PDB ID: 4TKP), SPRY (PDB ID: 2LM3), B-box and coiled-coil domains (PDB ID: 4TN3), available in the Protein Data Bank (www.rcsb.org). Missing amino acid backbone were built using the Modloop server^55^ and missing side chains were built using SCWRL4^56^ to generate an atomic model of full-length TRIM5α. Two-fold symmetry was imposed on the monomer and aligned with a partially resolved structure of the dimer (PDB ID: 4TN3).

The coarse-grained (CG) model was constructed by mapping five amino acid residues into one CG particle using methods similar to ones we have previously published^25–27^. Secondary structure elements within each distinct protein domain (RING: resid 1–81; SPRY: resid 286–497) were constrained as rigid bodies, and regions within the coiled coil domain dimer were also held as rigid bodies (Coiled-coil: resid 91–161, 166–216, and 221–281). TRIM5α proteins were modeled as dimers, since the dimer is significantly more stable than the monomer. TRIM5α are primarily dimeric in solution^57^ in part due to the extensive network of coiled-coil interactions across the length of the protein and has not been found to crystallize nor bind to CA as a monomer. Flexibility in the linker regions between the RING and B–box domains was added using harmonic bonds between neighboring CG particles (resid 81–91, *K*_bond_ = 10 kcal mol^−1^ Å^−2^). Similarly, flexibility in the SPRY and coiled-coil regions, and within the coiled-coil domain were maintained using a primitive elastic network model (ENM) that connected two adjacent groups of CG particles within a cutoff distance, *r*_cut_, with flexible harmonic bonds; the potential energy of the each bond is:

1 $$U_{{\mathrm{Bond}}} = K_{{\mathrm{bond}}}(r - r_0)^2$$

where *r* is the separation distance, and *r*_0_ is the equilibrium bond length, set to the distance found in the crystal structure (SPRY–CC resid 286–291, resid 176–202; *r*_cut_ = 17.8 Å; *K*_bond_ = 2.5 kcal mol^−1^ Å^−2^, and CC resid 156–161, 226, 266, resid 166–171, 216–221, 271; *r*_cut_ = 30 Å; *K*_bond_ = 0.5 kcal mol^−1^ Å^−2^). The spring constants for the bonded interactions at the interface of the RING domain and coiled-coil region were chosen to mimic an unstructured polymer chain, and the spring constants for the ENM at the interface between the coiled-coil region and SPRY domain were chosen to maintain non-bonded hydrophobic and electrostatic interactions between the L2 linker region and the SPRY domain^58^. Varying the spring constants for both interactions by a factor of ten did not significantly change the assembly behavior. Flexibility in the coiled-coil region however was necessary for the lattice to adapt to the varying curvatures in the capsid. Initial models that contained rigid coiled-coil domains formed flat 2D hexagonal arrays. The spring constants for the ENM in the coiled-coil region were lowered from 1.5 kcal/mol to 0.5 kcal/mol in increments of 0.25 kcal/mol until the lattice was observed to wrap around the capsid core. To maintain the overall CG protein shape, each CG particle included excluded volume interactions, using a soft cosine potential *U*_Vol_ given by:

2 $$U_{{\mathrm{Vol}}} = A\left[1 + \cos \left(\frac{{\pi r}}{{r_c}}\right)\right]$$

when *r* < *r*_*c*_. The cutoff (*r*_*c*_), the onset of excluded volume repulsion, between CG particles was set to the separation distances found in the crystallographic fragments of TRIM5α or the trimer-of-dimers interface if the distances were less than 10 Å, or otherwise 10 Å, since CG particles at this resolution have an average radii of 5 Å. *A* = 100 kcal/mol. The mass of the protein (57.31 kDa) was evenly distributed among the CG beads.

Non-bonded protein–protein interactions between TRIM5α dimers were added on the basis of X-ray structures of truncated TRIM5α RING, B-box, Coiled-coil and SPRY domains (mini RBCCs, PDB ID: 5IEA) that crystallized in the trimer-of-dimers configurations to mimic the alternating electrostatic, hydrophobic, and electrostatic contacts at the B-box interface. Full-length CG TRIM5α models were fit into the electron density to construct the trimer-of-dimers structure, and virtual CG particles for each monomer were added to the relative positions of the following residues on an adjacent TRIM5α monomer (resid 91–106, 121–151, 231–241, and 256–261). Each of the virtual particles was allowed to tether to any CG particle that represented the same residue used to generate its position, using an attractive Gaussian potential given below:

3 $$U_{{\mathrm{Int}}} = - \varepsilon \;e^{ - B(r - r_0)^2}$$

where *ε*_P_ = 0.7 kcal/mol is the well-depth, *B* = 0.01 Å^−2^ is the inverse interaction length, *r*_0_ = 0 Å, and *r* is the distance between the virtual particle and a CG particle that represented the same residue.

Non-bonded protein–protein interactions between the SPRY domain and CA were added on the basis of experimental mutagenesis studies^21,29^. Attractive interactions were assigned between CA residues (resid 83–132) that when mutated demonstrated impaired binding of TRIM5α and the variable loops of the SPRY domain (resid 326–351, 381–396, and 421–426) using the same Gaussian potential as the TRIM5α–TRIM5α dimer interaction with *ε*_C_ = 0.28 kcal/mol, and *B* = 2 Å^−1^, *r*_0_ = 20 Å. Contributions from non-specific interactions (e.g. dispersion forces) were assumed to be small enough to be negligible in this CG model.

### CG capsid models

Regular polygons were fit into electron cryo-tomography densities derived from intact virions^30^. Atomic models for the CA protein were then constructed from x-ray structures of the hexameric and pentameric capsomere structures (PDB ID: 3MGE, PDB ID: 3P05). Missing backbone and sidechain residues were built using Modloop and SCWRL4, and the model was subsequently coarse-grained at a resolution of 1 CG bead per 5 amino acid residues. To generate initial models of the capsid, the CG model was fit to the Cartesian coordinates and relative orientations of the polygons. The initial model was relaxed using a primitive elastic network model between C-terminal dimer contacts (resid 178–194, resid 178–194; *r*_cut_ = 20 Å; *K*_bond_ = 0.1 kcal mol^−1^ Å^−2^), and a brief 20 ps CG molecular dynamics simulation.

### MD simulations

All CG molecular dynamics simulations used the large-scale atomic/molecular massively parallel simulator (LAMMPS)^59^. In all cases, a temperature of 300 K was maintained with a Langevin thermostat. Solvent was treated implicity during Langevin dynamics, with a damping constant (*t*_damp_ = 200 ps) and included a frictional coefficient of (*m*/*t*_damp_) in the equations of motions to dampen sensitivity to protein–protein interactions, where *m* is the mass of a CG particle. A CG MD timestep of 200 fs was chosen, as the largest possible timestep without noticeable energy drift in the microcanonical (constant NVE) ensemble. The energy drift during test and production simulations is shown in Supplementary Figs. 4 and 5. Time integration was performed with the velocity Verlet algorithm. Simulation systems were initialized by placing TRIM5α subunits, uniformly distributed along the (*x*, *y*, *z*) dimensions and 150 Å from any capsid protein domain residues, in random orientations. Periodic boundary conditions were imposed on a simulation box centered on the center-of-mass of a CG HIV-1 capsid. The simulation box size was chosen as 0.064 μm^3^ to mimic a concentration of 3.2 μM, similar to that used in in vitro assembly experiments (0.25–5 μM)^16–18^. In total, each simulation consisted of ~1500 proteins. Similar to prior studies^25–27^, we report “CG time” in units of *τ* = 250,000 CG timesteps for all trajectories because of the well-known separation between CG and real-time dynamics^60,61^. Each system was run until lattice growth plateaued (~2.1 × 10^9^ CG timesteps).

### Diffusivity calculations

The diffusivities and mean-square displacements were computed for the center-of-geometry for residues in the B–box domain (resid 259–263). Statistics were collected across all assembly simulations for the six capsid morphologies and CA tube (~59,500 *τ*), and displacements were measured for each lag time in increments of *τ*. The MSD profiles for dimer populations in bulk solvent were fit to: MSD(*t*) = d*t*; whereas the MSD profiles for dimers on the capsid, or lattices on the capsid were fit to: ${\mathrm{MSD}}\left( t \right) = \frac{{{\mathrm{{d}}}t(s - e^{ - \lambda t})}}{{{{\mathrm{d}}} {t}\; + \;{s}\; -\; {e}^{ - \lambda t}}}$, which approaches d*t* for *t* << 1 and *s* − *e*^−*λt*^ for *t* >> 1 with *s* = MSD(*t* = ∞), and subsequently normalized by the MSD(*t* = 10*τ*). Monomers/dimers were assigned as bound to the capsid if any CG bead was within 40 Å of any CA particle. Monomers/dimers were assigned to form lattice contacts if any B–box domain residue was within 30 Å of a B-box domain residue belonging to another monomer/dimer. Monomers/dimers were considered to be free if they were not bound and did not form lattice contacts.

### Pair correlation function calculations

Pair correlation functions, *g*(*r*), were computed for the center-of-geometry of residues in the B–box domain (resid 259–263). Statistics were collected for frames in the assembly simulations for the 6 capsid morphologies and CA tube that contained hexameric or pentameric structures (~55,000 *τ*). Our CG models for TRIM5α and CA were flexibly fit into the experimental cryo-ET densities (Fig. 4b) to generate a snapshot for which the *g*(*r*) was also calculated. All calculations used a 0.5 Å bin width and 200 nm cutoff.

### SPRY–CA binding interface

CA residues within 20 Å of the SPRY domain were tabulated for all frames in the assembly simulations for the 6 capsid morphologies and CA tube (~59,500 *τ*) in increments of *τ*, and subsequently normalized to produce a contact probability distribution. The same calculation was performed for the CG model derived from the experimental cryo-ET density of TRIM5α associated with the helical CA tube, containing 2620 TRIM5α monomers. 3D volumetric densities were computed for the CG model of CA and then colored according to the contact probability values of each CG bead.

### Electron cryotomography and lattice mapping

TRIM5α coated HIV-1 CA tubes were prepared by co-incubation of the purified proteins^17,18^. The sample was mixed with equal volume of 10 nm BSA Gold Tracer (Electron Microscopy Sciences) and then applied on glow-discharged C-flat grids (Protochips) and plunge-frozen into liquid ethane. Cryo-tomograms were acquired using an FEI Titan Krios electron microscope operating at 300 kV and equipped with a Falcon II camera. Tilt series were collected using the data collection software Tomography 3.0 (FEI) with an angular range of −60° to +60°, angular increment of 1°, and pixel (px) size of 2.92 Å. Tomogram reconstruction was performed using the weighted back-projection approach in IMOD^62^. Weighted back-projection was used to reconstruct tomograms, and the contrast transfer function (CTF) was applied in IMOD. Lattice mapping was performed using the sub-tomogram averaging algorithms in the Dynamo software package^63^.

### Reporting summary

Further information on research design is available in the Nature Research Reporting Summary linked to this article.

## Supplementary information

Supplementary Information

Peer Review

Reporting Summary

Description of Additional Supplementary Files

Supplementary Movie 1

Supplementary Movie 2

## Data Availability

All data generated and used in this study, including the coarse grained-models, are available at https://github.com/alvinyu33/trim5-public/ or upon request to the corresponding author. The source data underlying Figs. 5a and 6a are provided as a Source Data file.

## Source Data

Source Data

## Code availability

All code generated and used in this study are available upon request to the corresponding author, Gregory A. Voth.
