# Supplementary material for: TRIM5α self-assembly and compartmentalization of the HIV-1 viral capsid
Source: Nat Commun. 2020 Mar 11;11:1307. doi: 10.1038/s41467-020-15106-1 (PMC7066149; doi:10.1038/s41467-020-15106-1)
Supplement: Supplementary file 1 — Supplementary Information [file 41467_2020_15106_MOESM1_ESM.docx]

**Supplementary Information**

TRIM5α Self-Assembly and Compartmentalization of the HIV-1 Viral Capsid

Yu et al.

**Corresponding Author:**

Gregory A. Voth

Email: [gavoth@uchicago.edu](mailto:gavoth@uchicago.edu)

Department of Chemistry

The University of Chicago

5735 S. Ellis Ave, SCL 123

Chicago, IL 60637

**Supplementary Note 1**

Production simulations were performed at the Department of Defense, Supercomputing Resource Center (DRSC) on the U.S. Army Engineer Research and Development Center (ERDC), Onyx machine, a Cray XC40/50 supercomputer. Each compute node was equipped with two Intel Xeon E5-2699v4 Broadwell processors, and a total of 44 cores per node. Each CG MD trajectory was simulated on a single node, using all 44 cores. A total of ~150 trajectories were simulated during parameter optimization (Fig. 3) at varying (ε_P_ , ε_C_) state points for ~2.1 × 10^9^ CG timesteps at a rate of: 61.6 × 10^6^ CG timesteps per day. Ideal parameters were chosen and used to perform assembly simulations on 5 different capsid morphologies (Fig. 7).

**
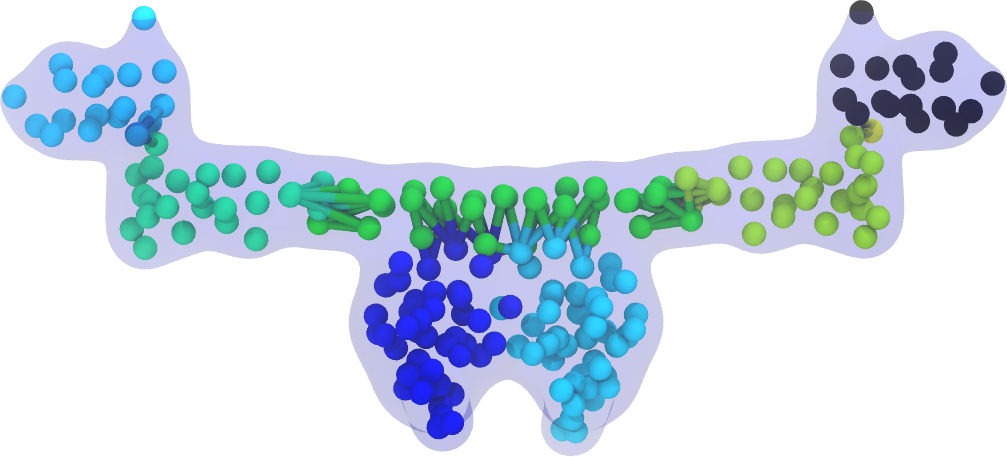
**

**Supplementary Figure 1**. A primitive elastic network connects different domains of the TRIM5α dimer. The positions and bonds between CG sites are shown above, colored by the protein domains, which are held rigid.

**
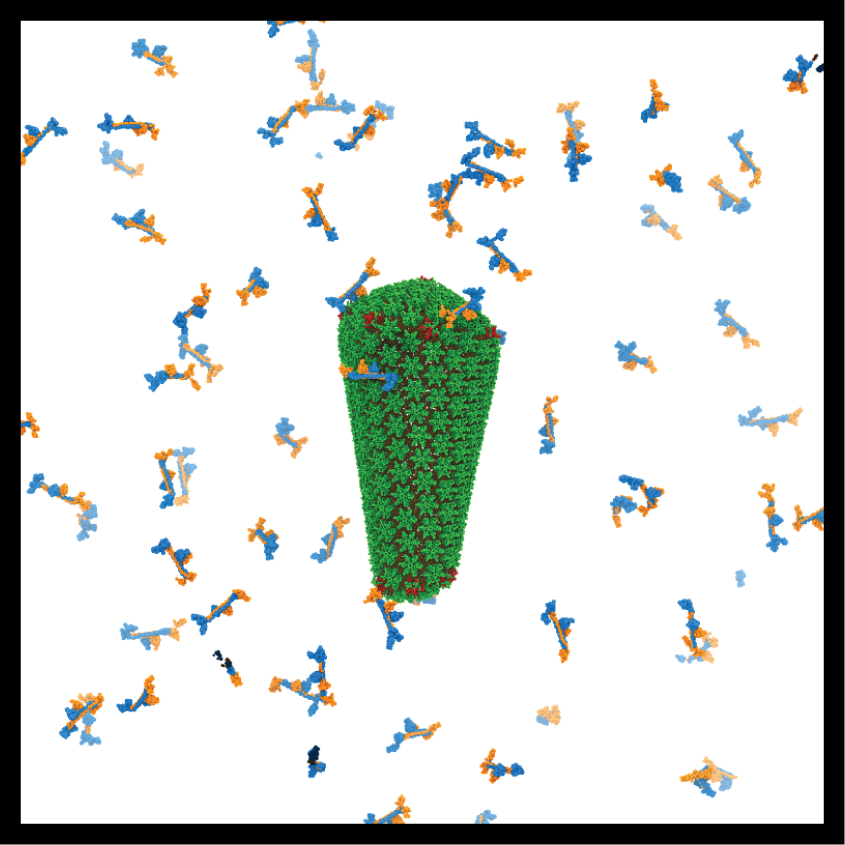
**

**Supplementary Figure 2.** Thermodynamic state point corresponding to $\varepsilon_{P}=0.7$, $\varepsilon_{C}=0$ kcal/mol for a CG assembly simulation after ~1000τ. Assembly of TRIM5α in solution does not occur in the absence of TRIM5α–CA interactions.

**
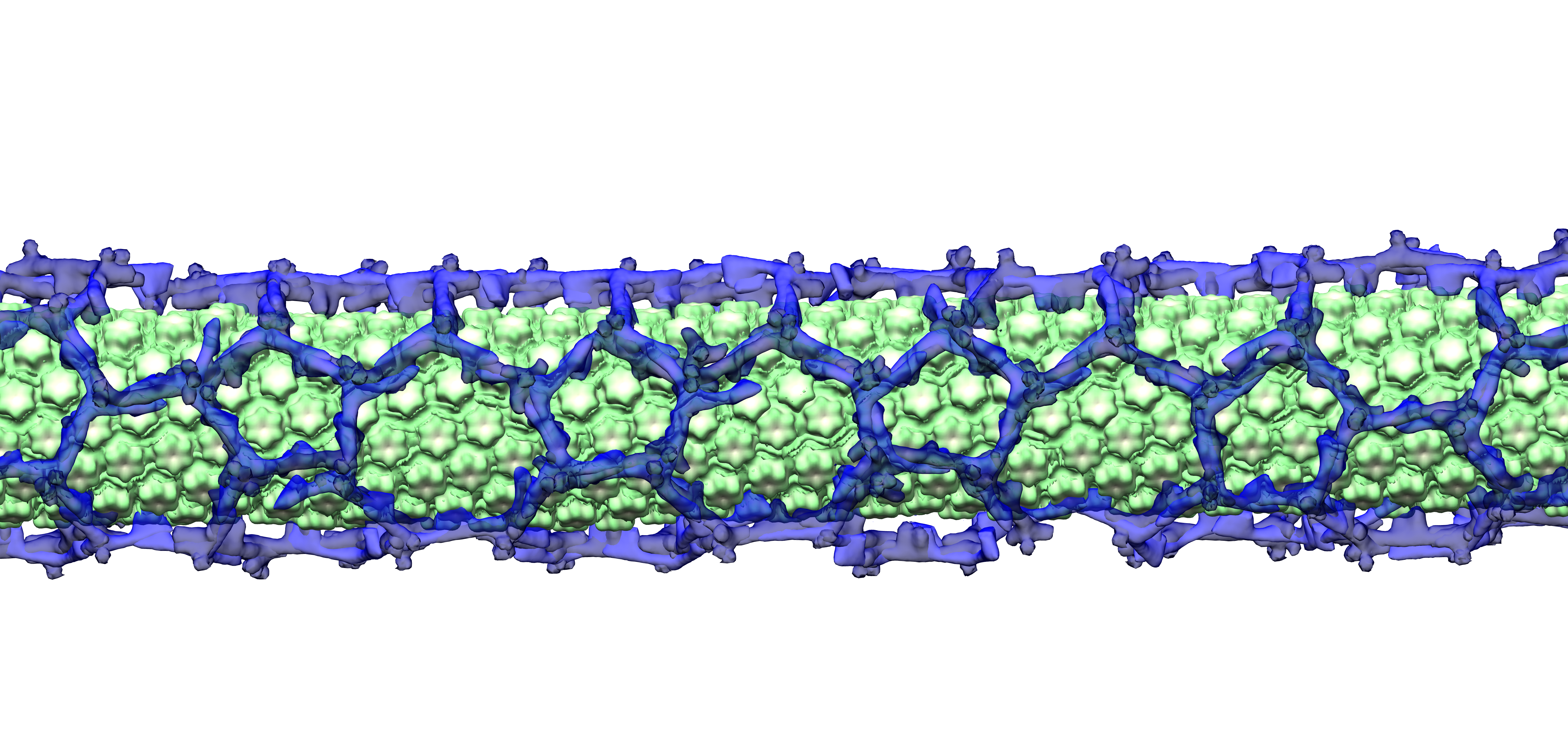
**

**Supplementary Figure 3.** Grain boundary in the TRIM5α lattice.


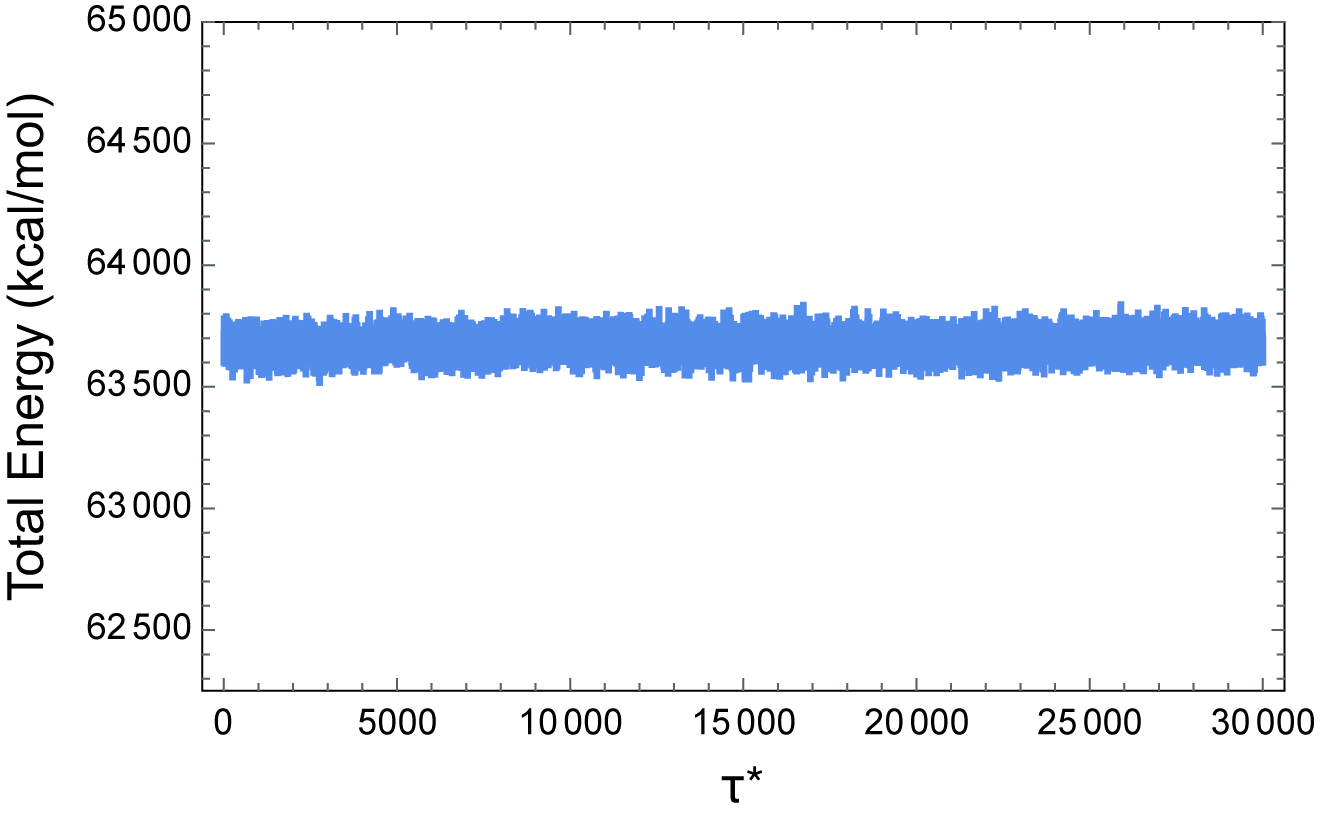


**Supplementary Figure 4.** The total energy as a function of the simulation time ($\tau^{*}=1000$CG MD timesteps) shows the energy drift for a Langevin dynamics test run (NVT ensemble) with a CG MD timestep of 200 fs.

**
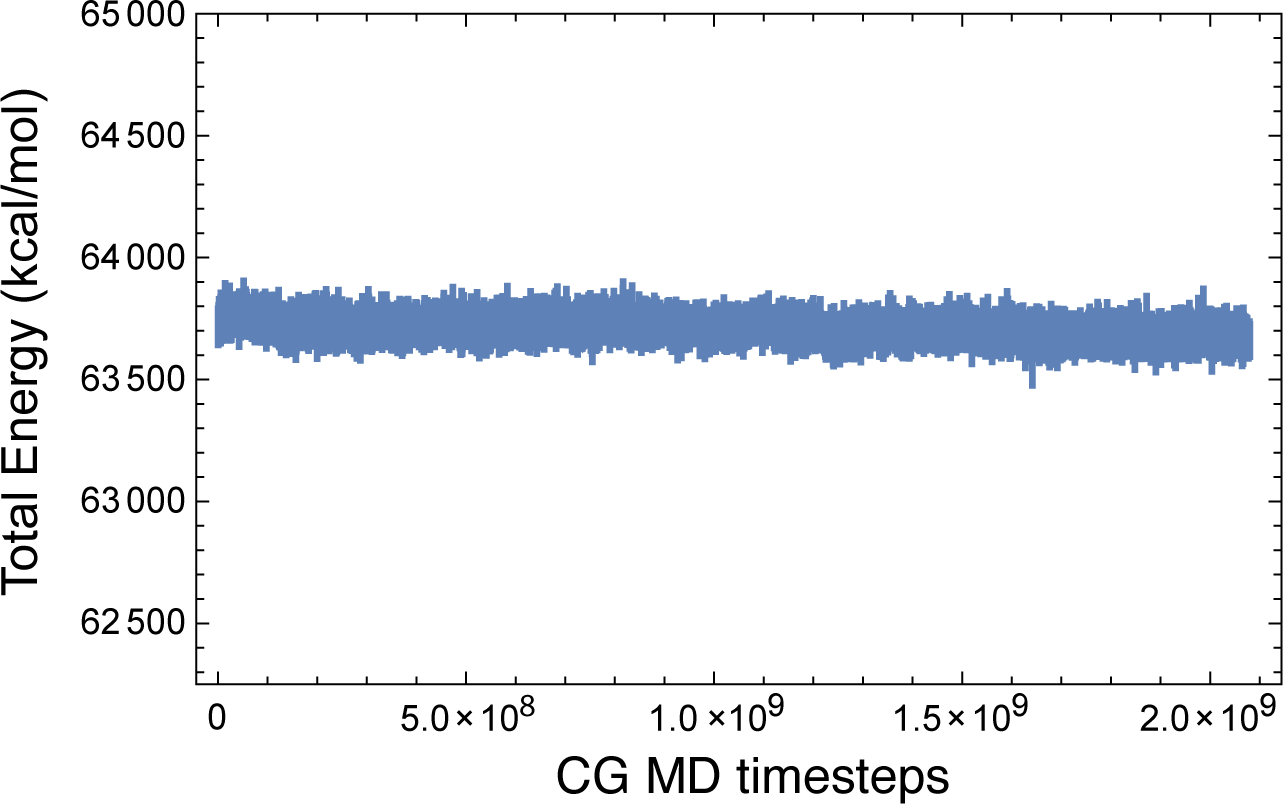
**

**Supplementary Figure 5.** The total energy as a function of the simulation time shows the energy drift during a Langevin dynamics production run (NVT ensemble) with a CG MD timestep of 200 fs.

**Source Data File**

Figure5.txt: “Pair correlation functions, g(r), for the structure of the CG hexamer, pentamer, and experiment.”

The raw data for the pair-correlation functions calculated in Figure 5A.

Figure6.txt: “CA domain residues contacting the SPRY domain”

The raw data for the residues contacting the SPRY domain in Figure 6A from both experiment and simulation.
