# Supplementary material for: TRIM5α self-assembly and compartmentalization of the HIV-1 viral capsid
Source: Nat Commun. 2020 Mar 11;11:1307. doi: 10.1038/s41467-020-15106-1 (PMC7066149; doi:10.1038/s41467-020-15106-1)
Supplement: Supplementary file 4 — Description of Additional Supplementary Files [file 41467_2020_15106_MOESM4_ESM.docx]

**Description of Additional Supplementary Files**

File name: Supplementary Movie 1
Description: TRIM5α encages the HIV-1 capsid core

File name: Supplementary Movie 2
Description: TRIM5α diffuses on the capsid surface
