# Supplementary material for: TRIM5α self-assembly and compartmentalization of the HIV-1 viral capsid
Source: Nat Commun. 2020 Mar 11;11:1307. doi: 10.1038/s41467-020-15106-1 (PMC7066149; doi:10.1038/s41467-020-15106-1)
Supplement: Supplementary file 2 — Peer Review [file 41467_2020_15106_MOESM2_ESM.pdf]

Reviewers' comments:

Reviewer #1 (Remarks to the Author):

Yu et al: TRIM5a self-assembly and compartmentalization of the HIV-1 Viral Capsid.

The authors have determined here energetic criteria and dynamics for the successful assembly of TRIM5a proteins around HIV capsids of varying shapes. The paper combines coarse-grained Langvein dynamics simulations with cryoET to predict the structures of TRIM5a lattices on the capsid, and the pathways through which they assemble. They make several somewhat surprising but ultimately reasonable and justified findings about the assembly, including that the lattice accommodates several geometries of polygons, not just hexagons, that the lattice dynamically remodels, including in response to changes in surface curvature, and that the lattice moves on the surface. They are able to directly compare the results from the simulations to the experiments through structural metrics. The agreement is relatively good. The system studied is significant, as these types of proteins are important in the immune response to HIV infection in a number of animals. The paper is well-written, the results are significant, Figures are clear, and the conclusions are justified: recommend to accept with revisions.

A few points:

1. They find that successful assembly seems to require both the TRIM-TRIM interactions, and the interactions with the surface, that localizes the proteins and helps stabilize binding between the dimers into trimers and lattices. However, there are point missing from their phase diagram of epsP/epsC values that would make this point more strongly. In particular, at the optimal value of epsP=0.7, do the TRIM5a assemble in solution at this concentration (i.e. with epsC=0)? This state point would more convincingly address whether the encapsulation requires the capsid surface to reduce the search space and trigger the assembly. The opposite extreme may not be necessary (epsC=0.3 and epsP=0) because the phase diagram does already seem to show that the optimal surface interaction strength of epsC=0.3 is not strong enough to stabilize TRIM on the surface by itself.
2. Similar to the above, are their results consistent with a >1mM interaction of the proteins with the surface?
3. It seems like the TRIM5a dimer cannot dissociate into monomers, due to the ENM. Is this a reasonable assumption for the monomer behavior, that dimers are so much more stable? This is not clearly commented on. On a related note, it is somewhat confusing in Fig 2 how the N(t) tracks contacts between monomers and the surface. So the point of the inset is to show that usually, when a dimer sticks to the surface, both monomers make a contact, but sometimes only one makes a contact at a time?
4. For the diffusion or hopping on the surface by the single TRIM5a, there is a maximal displacement from any starting point due the geometry of the surface. Does the RMSD calculation account for the net distance traveled on the surface, even if the position can return back to where it started by fully looping around the circumference?
5. In the model, are the only interactions between TRIM-TRIM or TRIM-CAPSID excluded volume, except the two Gaussian potentials? So the diffusion/hopping on the surface is entirely mediated by the weak contacts between the TRIM and the CA domains, no nonspecific interactions?
6. Aren't the sites of interaction between SPRY and CA determined by their epsC parameterized interactions? Which residues of the CA domains have the attractive region for the SPRY, are they the same that show up in the plot Fig 6? It would be beneficial for this section for the authors to clarify that despite some contacts being parameterized to be attractive, other residues (I assume) nonetheless show preferential contact, presumably due to sterics or excluded volume, if there are no nonspecific interactions. They mention that mutagenesis was used to define where on SPRY it binds to

CA, but they did not discuss where on CA it binds to SPRY or why.
7. Figure 6B/C legends need more description, especially C.
8. Only one of the movie files works. Can we not see a movie of the assembly process?

Reviewer #2 (Remarks to the Author):

Review of “TRIM5α Self-Assembly and Compartmentalization of the HIV-1 Viral Capsid”

This manuscript describes coarse-grain dynamics simulations of the TRIM5a dimer assembling and encaging the intact HIV Capsid core particle. It goes on to analyze the nature of the process as shown by the simulations and validation of some of the observations by comparison to experimental cryo-electron tomography. This work represents a significant extension of dynamics simulation in understanding biological assembly processes, it also presents a mechanistic hypothesis for the action of TRIM5 as a host restriction factor in HIV infection and is worthy of the wide readership of Nature Communications.

The coarse grain model of the TRIM5a dimer and the dynamic assembly simulations, themselves, represent a state of the art molecular dynamics application for a large heterogeneous system with over 1500 flexible multi-domain protein dimers of TRIM5a interacting with a very large Capsid Core molecular assembly. Each of the simulation runs needed over 2 billion time steps of this size system. Key findings from the simulations demonstrate both the dynamic equilibrium of the assembly process, involving continual making and breaking of protein interactions, as well as the nature the diffusive processes of the assembly components on the surface of the target capsid particle. Analysis also helps elucidate the nature and mapping of the non-specific interactions of the TRIM5a SPRY domain interactions with the Capsid assembly.

The model for the TRIM5a dimer implements limited flexibility between the domains of the proteins in the complex and is the fundamental element in the simulation. The domain interfaces are modeled by a series of springs (flexible harmonic bonds) between the spheres representing successive groups of 5 amino acids. The spring constants for these interactions, K, are listed for the various interfaces. The authors have explored variations of these constants, and have indicated that changes by a factor of 10 had no significant impact on the results of the simulations. They have also made available the atomic level homology model of the TRIM5a dimer from which the coarse grained model was built. This information will be helpful for duplicating their results. In that vein it would be instructive to have more information (in the supplementary material), as to the computational resources that were needed for this study. How much computer time was used? Were GPUs or other parallel hardware utilized?

This manuscript is a significant contribution to both the field of large mesoscale biological simulation, and to our understanding of assembly processes in the field of HIV biology. This reviewer believes that it would be of interest to the wide readership of Nature Communications and is worthy of publication.

Arthur Olson

Reviewer #3 (Remarks to the Author):

Abstract
This work investigates the structure and dynamics of assembled TRIM5a around an HIV capsid by coarse-grained (CG) simulations. From experiments, TRIM5a proteins form a hexgonal lattice structure surrounding an HIV capsid, leading to the resistance of HIV infection. Thus, to understand the mechanism of TRIM5a encase is useful to inhibit HIV infection. Moreover, the binding interaction between TRIM5a and capsid unit is very weak from equilibrium constant. The role of such weak interaction is of special interest for fundamental self-assembly. Their CG simulation provides two results. One is that the interaction strength of TRIM5a-capsid and TRIM5a-TRIM5a is substantially balanced to form the above lattice structure around HIV. The other is that the formed lattice structure has defects in the hexagonal lattice, which is difficult to be observed in low-resolution experimental data. In summary, their extensive CG simulation elucidates the fine structure of the enclosed HIV capsid by a TRIM5a lattice cage, its dynamics of surface diffusion, and the reason for weak binding interaction between TRIM5a and capsid domain.
While their work provides the fundamental insight of self-assembled structure and dynamics of the proteins, there are following questions for the draft.
Major points
• In the coarse-graining model, how solvent molecule is treated? I could not also find solvent treatment in cited Ref. 56. Next, if the solvent is treated implicitly, they need to discuss the possible effect for the self-assembled structure because the simulation results are sensitive to small interaction energy function change, and, hence, solvent interaction is considered to be a large impact on the result. What does the interaction of water/ion-TRIM5a water/ion-CA influence this result?
• In the method section, the time step was employed at 200 fs. However, in previous work of CG-simulation in Ref. 30, the time step was at 10 fs, twenty times larger than the reference work of the CG-model. In the draft, they write that the energy drift is small enough at 200fs. However, energy drift depends on the algorithm. They first must write which numerical algorithm is used for time propagation for both NVE and NVT. Then, they also need to show the energy drift of both the NVE and self-assembly NVT simulation in Supporting information. Moreover, because they employed an NVT ensemble, how did they determine the box size of simulation and resulting density? Such information is necessary for readers to understand the simulation accuracy.
• Simulations assume that the self-assembly of TRIM5a forms under the complete HIV capsid. On the other hand, if an HIV virus is active, the replication of HIV continues to occur in human body. In this case, self-assembly of both HIV and TRIM5a seems to occur at the same time. Is such a possibility excluded for the purpose of blocking HIV by TRIM5a?
• In this work, they found the existence of optimal interactions of TRIM5a-capsid and TRIM5a-TRIM5a to encase the HIV capsid properly. Does the optimal parameter correspond to the weak affinity, Kd, characterized by experiments? We are interested to know the binding affinity characterized by free energy, not the simple potential energy.
• This work emphasizes the existence of defects in the hexagonal structure of TRIM5a lattice. However, the existence of defects in a crystal (ordered structure) is rather ordinary in nature; for example, it often prevents the protein structure determination from X-ray diffraction and efficient semiconductor pro-

duction in an industry. Thus, if they emphasize the defects, they need to propose how these defects are useful for the purpose, such as resistance to virus infection. However, such a description is not seen in this draft so far. For example, in the Ref 30, they refer to the influence of defects in the lattice structure. It seems that such a discussion should be critical.

Minor points

- On page 3, ”human -immunodeficiency virus type 1 (HIV-1)” is used two times in lines 9 and 11. The second one is redundant.
- On page 3, ”several studies, including ours, have suggested that the weak interactions. . . the capsid surface23” refers to ”several” but the number of references is only one, Ref 23. Correct or add the sentence or the citation.
- On page 6, in the word of ”how the number of lattice-forming monomers, N(τ),” how do you count the number of forming monomer or free monomer? Such value depends on the definition.
- On page 14, ”Regular polygons were fit into electron cryo-tomography densities derived from intact virions.”. How is this regular polygons fitting done? Ref 30 shows only the intact structures, and no polygon fitting appears.
- In Figure 6 caption ”(B) The simulated contact probability is mapped back onto the atomic structure of the CA domain.” Does this back mapping means the method part of ”Electron cryotomography (cryo-ET) and lattice mapping,” or how did you map the contact probability?

Conclusion

In conclusion, their extensive coarse-grain simulations provide significant insight into the fine atomic structure of TRIM5a surrounding the HIV capsid structure and the diffusion dynamics of TRIM5a on the capsid surface during the self-assembly. On the other hand, there are the above points to be improved in the draft before publication.

Reviewer #1:

Yu et al: TRIM5a self-assembly and compartmentalization of the HIV-1 Viral Capsid.

The authors have determined here energetic criteria and dynamics for the successful assembly of TRIM5a proteins around HIV capsids of varying shapes. The paper combines coarse-grained Langvein dynamics simulations with cryoET to predict the structures of TRIM5a lattices on the capsid, and the pathways through which they assemble. They make several somewhat surprising but ultimately reasonable and justified findings about the assembly, including that the lattice accommodates several geometries of polygons, not just hexagons, that the lattice dynamically remodels, including in response to changes in surface curvature, and that the lattice moves on the surface. They are able to directly compare the results from the simulations to the experiments through structural metrics. The agreement is relatively good. The system studied is significant, as these types of proteins are important in the immune response to HIV infection in a number of animals. The paper is well-written, the results are significant, figures are clear, and the conclusions are justified: recommend to accept with revisions.

We thank the reviewer for the careful and supportive assessment of our manuscript and have modified our manuscript accordingly. We hope that our manuscript has been satisfactorily improved.

A few points:

1. They find that successful assembly seems to require both the TRIM-TRIM interactions, and the interactions with the surface, that localizes the proteins and helps stabilize binding between the dimers into trimers and lattices. However, there are point missing from their phase diagram of epsP/epsC values that would make this point more strongly. In particular, at the optimal value of epsP=0.7, do the TRIM5a assemble in solution at this concentration (i.e. with epsC=0)? This state point would more convincingly address whether the encapsulation requires the capsid surface to reduce the search space and trigger the assembly. The opposite extreme may not be necessary (epsC=0.3 and epsP=0) because the phase diagram does already seem to show that the optimal surface interaction strength of epsC=0.3 is not strong enough to stabilize TRIM on the surface by itself.

   This is an excellent point. We have added a figure in the SI (Supplementary Fig. 3) for the $\varepsilon_\text{P} = 0.7$, $\varepsilon_\text{P} = 0$ kcal/mol state point, and discussion in the main text on pg. 8. At this state point and concentration, some dimers transiently form dimer-of-dimer structures, but TRIM5α does not assemble into large-scale lattices.

2. Similar to the above, are their results consistent with a >1mM interaction of the proteins with the surface?

   Our simulations are consistent with a very weak affinity (> 1 mM) for the protein–capsid interaction. Quantitatively predicting the binding free energy between TRIM5α and CA, however, would be most accurately assessed using detailed atomistic MD simulations, which we are actively pursuing in a separate study.

3. It seems like the TRIM5a dimer cannot dissociate into monomers, due to the ENM. Is this a reasonable assumption for the monomer behavior, that dimers are so much more stable? This is not clearly commented on. On a related note, it is somewhat confusing in Fig 2 how the N(t) tracks contacts between monomers and the surface. So the point of the inset is to show that usually, when a dimer sticks to the surface, both monomers make a contact, but sometimes only one makes a contact at a time?

   Indeed, TRIM5α dimers are much more stable than monomers, and TRIM5α exists primarily as dimers in solution (Langelier CR et al, J.Virol, 2008), in part due to the extensive network of coiled-coil interactions across the length of the protein. TRIM5α does not crystallize nor bind to CA in the monomeric form. We have updated this assumption in the main text on pg. 13.

   We apologize for the confusion. N(t) tracks the number of TRIM5α monomers forming lattice contacts with other TRIM5alpha monomers, while on the surface of the capsid. The point of the inset was to show that these lattice contacts break (negative dN) and form (positive dN) during assembly. This is now clarified in the Fig 2 legend.

4. For the diffusion or hopping on the surface by the single TRIM5a, there is a maximal displacement from any starting point due the geometry of the surface. Does the RMSD calculation account for the net distance traveled on the surface, even if the position can return back to where it started by fully looping around the circumference?

   This is a good point. Generally periodicity (e.g., on the 2D surface of the capsid) should be taken into account in such a calculation, but the lag times examined were small enough for the protein to not diffuse around the circumference of the capsid.

5. In the model, are the only interactions between TRIM-TRIM or TRIM-CAPSID excluded volume, except the two Gaussian potentials? So the diffusion/hopping on the surface is entirely mediated by the weak contacts between the TRIM and the CA domains, no nonspecific interactions?

   This is correct. Contributions from non-specific interactions arising from, e.g., dispersion forces were assumed to be small enough to be negligible in the CG model. In prior models, we used a soft-core Lennard Jones potential, which included non-specific interactions, but this did not significantly affect the assembly behavior. We have added discussion on pg. 14.

6. Aren't the sites of interaction between SPRY and CA determined by their epsC parameterized interactions? Which residues of the CA domains have the attractive region for the SPRY, are they the same that show up in the plot Fig 6? It would be beneficial for this section for the authors to clarify that despite some contacts being parameterized to be attractive, other residues (I assume) nonetheless show preferential contact, presumably due to sterics or excluded volume, if there are no nonspecific interactions. They mention that mutagenesis was used to define where on SPRY it binds to CA, but they did not discuss where on CA it binds to SPRY or why.

The residues of CA domains that have attractive interactions for the SPRY domain are resids 83–132, and they appear as two of the largest peaks for the contact probability in Fig 6. The contact probabilities for residues are determined by a combination of sterics, excluded volume, sites that facilitate TRIM5α lattice contacts, and the attractive TRIM5α–CA potential. We do find it interesting that many sites not directly parameterized show preferential contact and agree rather well with the experimental contacts. We have added discussion on pg 10 and the residue identities on pg 14.

7. Figure 6B/C legends need more description, especially C.

We have included additional description in the Figure 6 B/C legends.

8. Only one of the movie files works. Can we not see a movie of the assembly process?

We noticed the original SI movie 1 was not encoded with Y'UV 4:2:0 planar chroma subsampling, required by video players such as QuickTime and Windows Media Player. A new SI movie 1 has been uploaded.

Reviewer #2:

Review of "TRIM5α Self-Assembly and Compartmentalization of the HIV-1 Viral Capsid"

This manuscript describes coarse-grain dynamics simulations of the TRIM5a dimer assembling and encaging the intact HIV Capsid core particle. It goes on to analyze the nature of the process as shown by the simulations and validation of some of the observations by comparison to experimental cryo-electron tomography. This work represents a significant extension of dynamics simulation in understanding biological assembly processes, it also presents a mechanistic hypothesis for the action of TRIM5 as a host restriction factor in HIV infection and is worthy of the wide readership of Nature Communications.

We thank the reviewer for an excellent review of the manuscript.

The coarse grain model of the TRIM5a dimer and the dynamic assembly simulations, themselves, represent a state of the art molecular dynamics application for a large heterogeneous system with over 1500 flexible multi-domain protein dimers of TRIM5a interacting with a very large Capsid Core molecular assembly. Each of the simulation runs needed over 2 billion time steps of this size system. Key findings from the simulations demonstrate both the dynamic equilibrium of the assembly process, involving continual making and breaking of protein interactions, as well as the nature the diffusive processes of the assembly components on the surface of the target capsid particle. Analysis also helps elucidate the nature and mapping of the non-specific interactions of the TRIM5a SPRY domain interactions with the Capsid assembly.

The model for the TRIM5a dimer implements limited flexibility between the domains of the proteins in the complex and is the fundamental element in the simulation. The domain interfaces are modeled by a series of springs (flexible harmonic bonds) between the spheres representing

successive groups of 5 amino acids. The spring constants for these interactions, K, are listed for the various interfaces. The authors have explored variations of these constants, and have indicated that changes by a factor of 10 had no significant impact on the results of the simulations. They have also made available the atomic level homology model of the TRIM5a dimer from which the coarse grained model was built. This information will be helpful for duplicating their results.

9. In that vein it would be instructive to have more information (in the supplementary material), as to the computational resources that were needed for this study. How much computer time was used? Were GPUs or other parallel hardware utilized?

   We have added additional informational in the supplementary material as to the computational resources and computer time used in this study. We did not use GPUs, but we did use a highly parallel Cray XC50 machine. In general, assembly simulations took about a month from start to finish on 150 nodes, using 44 CPU cores per node.

This manuscript is a significant contribution to both the field of large mesoscale biological simulation, and to our understanding of assembly processes in the field of HIV biology. This reviewer believes that it would be of interest to the wide readership of Nature Communications and is worthy of publication.

Arthur Olson

We are grateful to the reviewer for both a thorough consideration of the manuscript and his insightful comments.

Reviewer #3:

Abstract
This work investigates the structure and dynamics of assembled TRIM5a around an HIV capsid by coarse-grained (CG) simulations. From experiments, TRIM5a proteins form a hexgonal lattice structure surrounding an HIV capsid, leading to the resistance of HIV infection. Thus, to understand the mechanism of TRIM5a encase is useful to inhibit HIV infection. Moreover, the binding interaction between TRIM5a and capsid unit is very weak from equilibrium constant. The role of such weak interaction is of special interest for fundamental self-assembly. Their CG simulation provides two results. One is that the interaction strength of TRIM5a-capsid and TRIM5a-TRIM5a is substantially balanced to form the above lattice structure around HIV. The other is that the formed lattice structure has defects in the hexagonal lattice, which is difficult to be observed in low-resolution experimental data. In summary, their extensive CG simulation elucidates the fine structure of the enclosed HIV capsid by a TRIM5a lattice cage, its dynamics of surface diffusion, and the reason for weak binding interaction between TRIM5a and capsid domain. While their work provides the fundamental insight of self-assembled structure and dynamics of the proteins, there are following questions for the draft.

We thank the reviewer for the positive comments and bringing these questions to our attention.

Major points

10. In the coarse-graining model, how solvent molecule is treated? I could not also find solvent treatment in cited Ref. 56. Next, if the solvent is treated implicitly, they need to discuss the possible effect for the self-assembled structure because the simulation results are sensitive to small interaction energy function change, and, hence, solvent interaction is considered to be a large impact on the result. What does the interaction of water/ion-TRIM5a water/ion-CA influence this result?

    Solvent dynamics was treated implicitly using Langevin dynamics, which includes a frictional term in the equations of motion to dampen sensitivity to protein–protein interactions as stated originally, but this could have been made clearer. Including protein–solvent interactions in CG models in a more rigorous fashion than the implicit treatment used in our work is an important and active area of research, but beyond the scope of our present paper. This has been clarified on pg. 15.

11. In the method section, the time step was employed at 200 fs. However, in previous work of CG-simulation in Ref. 30, the time step was at 10 fs, twenty times larger than the reference work of the CG-model. In the draft, they write that the energy drift is small enough at 200fs. However, energy drift depends on the algorithm. They first must write which numerical algorithm is used for time propagation for both NVE and NVT. Then, they also need to show the energy drift of both the NVE and self-assembly NVT simulation in Supporting information. Moreover, because they employed an NVT ensemble, how did they determine the box size of simulation and resulting density? Such information is necessary for readers to understand the simulation accuracy.

    We note that the CG time step employed in our more recent work referenced in the CG model (Ref. 26, Pak et al. *PNAS* Vol 114 Issue 47 pgs E10056-E10065) was also 200 fs. We have updated the Supporting Information with a figure for the energy drift for this time step (Supplementary Fig. 4). Additional simulation details, such as the algorithm for time propagation and choice of box size have been included on pg. 15. As is generally well-known, we also note that CG time is not the same as real (actual) time (the fomer is in general effectively much larger as appears in an integrator timestep, the latter being a technical detail of an algorithm).

12. Simulations assume that the self-assembly of TRIM5a forms under the complete HIV capsid. On the other hand, if an HIV virus is active, the replication of HIV continues to occur in human body. In this case, self-assembly of both HIV and TRIM5a seems to occur at the same time. Is such a possibility excluded for the purpose of blocking HIV by TRIM5a?

    Self-assembly of the capsid occurs extracellularly during the maturation process, within viral particles, whereas self-assembly of TRIM5α occurs in the cytoplasm, after virions have fused and deposited capsids into host cells.

13. In this work, they found the existence of optimal interactions of TRIM5a-capsid and TRIM5a-TRIM5a to encase the HIV capsid properly. Does the optimal parameter

correspond to the weak affinity, Kd, characterized by experiments? We are interested to know the binding affinity characterized by free energy, not the simple potential energy.

The optimal parameter does correspond to a very weak affinity; however, quantitatively predicting a free energy of binding between TRIM5α and CA would be best suited for detailed atomic simulations, which we are actively pursuing in a separate study.

14. This work emphasizes the existence of defects in the hexagonal structure of TRIM5a lattice. However, the existence of defects in a crystal (ordered structure) is rather ordinary in nature; for example, it often prevents the protein structure determination from X-ray diffraction and efficient semiconductor production in an industry. Thus, if they emphasize the defects, they need to propose how these defects are useful for the purpose, such as resistance to virus infection. However, such a description is not seen in this draft so far. For example, in the Ref 30, they refer to the influence of defects in the lattice structure. It seems that such a discussion should be critical.

This is a good point. We have added discussion on pg. 12.

Minor points

15. On page 3, ”human -immunodeficiency virus type 1 (HIV-1)” is used two times in lines 9 and 11. The second one is redundant.

We have deleted the second usage.

16. On page 3, ”several studies, including ours, have suggested that the weak interactions. . . the capsid surface23” refers to ”several” but the number of references is only one, Ref 23. Correct or add the sentence or the citation.

We have added a citation.

17. On page 6, in the word of ”how the number of lattice-forming monomers, N(τ),” how do you count the number of forming monomer or free monomer? Such value depends on the definition.

Free monomers were considered to be monomers that did not bind, nor form lattice contacts. Monomers were assigned to form lattice contacts if any CG bead representing the B–box domain residues was within 30 Å of the B–box domain residue of another monomer. This is clarified on pg 16.

18. On page 14, ”Regular polygons were fit into electron cryo-tomography densities derived from intact virions.”. How is this regular polygons fitting done? Ref 30 shows only the intact structures, and no polygon fitting appears.

This is in Mattei et al. *Science* 2016, Vol. 354, Issue 6318, pp. 1434-1437 (Ref 30) Figure 2A. The positions and orientations (Euler angles) of each hexamer from the cryo-EM data set were computed as described in that paper.

19. In Figure 6 caption "(B) The simulated contact probability is mapped back onto the atomic structure of the CA domain." Does this back mapping means the method part of "Electron cryotomography (cryo-ET) and lattice mapping," or how did you map the contact probability?

    We have added additional clarification in the Fig. 6B legend and methods section on pg. 16.

Conclusion

In conclusion, their extensive coarse-grain simulations provide significant insight into the fine atomic structure of TRIM5a surrounding the HIV capsid structure and the diffusion dynamics of TRIM5a on the capsid surface during the self-assembly. On the other hand, there are the above points to be improved in the draft before publication.

We thank the reviewer for the positive comments and bringing these points to our attention.

REVIEWERS' COMMENTS:

Reviewer #1 (Remarks to the Author):

Thorough and thoughtful responses to all referee reports, along with strong additional calculations. Very nice paper, accept as is.

Reviewer #2 (Remarks to the Author):

The authors have responded to the issues raised in the previous reviews and have modified the manuscript and supplemental materials to my satisfaction

Reviewer #3 (Remarks to the Author):

Report on:
" TRIM5 α Self-Assembly and Compartmentalization of the HIV-1 Viral
Capsid"

Authors' response to the previous review comments mostly answer questions, which could improve the quality of their draft.

The remaining point is that the time series of the potential energy during their PRODUCTION run is not attached in the new Supporting information. The NVE ensemble was newly attached. Although their analysis is done in detail, the qualification of simulation itself, such as energy conservation, is little in their draft. Because validiation of the CG model itself should be done before in the previous works, it is enough to add such information in the Supporting information.

Overall, their work can provide valuable insight regarding the encapsulation of HIV capsid by TRIM-5a and extend our knowledge for HIV.

Reviewer #1:

Thorough and thoughtful responses to all referee reports, along with strong additional calculations. Very nice paper, accept as is.

Reviewer #2:

The authors have responded to the issues raised in the previous reviews and have modified the manuscript and supplemental materials to my satisfaction

Reviewer #3:

Report on:
" TRIM5 α Self-Assembly and Compartmentalization of the HIV-1 Viral Capsid"

Authors' response to the previous review comments mostly answer questions, which could improve the quality of their draft.

> The remaining point is that the time series of the potential energy during their PRODUCTION run is not attached in the new Supporting information. The NVE ensemble was newly attached. Although their analysis is done in detail, the qualification of simulation itself, such as energy conservation, is little in their draft. Because validiation of the CG model itself should be done before in the previous works, it is enough to add such information in the Supporting information.
>
> We have updated the Supporting Information with the total energy time series during a production run.

Overall, their work can provide valuable insight regarding the encapsulation of HIV capsid by TRIM-5a and extend our knowledge for HIV.

We thank all reviewers for the positive and supportive comments.
